# Supplementary material for: Preventive Role of Diet Interventions and Dietary Factors in Type 2 Diabetes Mellitus: An Umbrella Review
Source: Nutrients. 2020 Sep 6;12(9):2722. doi: 10.3390/nu12092722 (PMC7551929; doi:10.3390/nu12092722)
Supplement: Supplementary file 1 [file nutrients-12-02722-s001.pdf]

Supplementary Table 1. Search terms and search strategies in PubMed

Domain	Search	Terms
**Intervention (I)**		
**I-1**	#1	lifestyle [MeSH term]
	#2	“lifestyle change”
	#3	“lifestyle modification”
	#4	“lifestyle adaptation”
	#5	“lifestyle intervention”
	#6	“lifestyle therapy”
	#7	“lifestyle treatment”
	#8	“behavior change”
	#9	“behavior modification”
	#10	“behavior adaptation”
	#11	“behavior intervention”
	#12	“behavior therapy”
	#13	“behavior treatment”
	#14	**#1 OR #2 OR #3 OR #4 OR #5 OR #6 OR #7 OR #8 OR #9 OR #10 OR #11 OR #12 OR #13**
**I-2**	#15	"non-pharmacological*"
	#16	"nonpharmacological*"
	#17	**#15 OR #16**
**I-3**	#18	"Diet, Carbohydrate-Restricted"[Mesh]
	#19	"Diet, Mediterranean"[Mesh]
	#20	"Diet, Fat-Restricted"[Mesh]
	#21	"Diet, Reducing"[Mesh]
	#22	"Diet Therapy"[Mesh]
	#23	#18 OR #19 OR #20 OR #21 OR #22
	#24	"diet* control"
	#25	“dietary control”
	#26	#24 OR #25
	#27	"low glycemic index"
	#28	"low carbohydrate"
	#29	"dietary approach to stop hypertension"
	#30	"healthy eat*"

	#31	"clean food"
	#32	#27 OR #28 OR #29 OR #30 OR #31
	#33	**#22 OR #26 OR #32**
**I-4**	#34	"physical activity"
	#35	Exercise
	#36	Sport
	#37	Workout
	#38	"work out"
	#39	#37 OR #38
	#40	"resistance training"
	#41	"strength training"
	#42	"weight training"
	#43	**#34 OR #35 OR #36 OR #39 OR #40 OR #41 OR #42**
**(I 1-4)**	#44	**#14 OR #17 OR #33 OR #43**
**Outcome (O)**	#45	prevent*
	#46	delay*
	#47	reduc*
	#48	#62 OR #63 OR #64
	#49	"Diabetes Mellitus, Type 2"[Mesh]
	#50	"diabetes mellitus"
	#51	#66 OR #67
	#52	**#48 AND #51**
**I + O**	#53	#44 AND #52
**(I + O) Filtered**	#54	#53 Filters: Systematic Reviews

Supplementary Table 2. Search terms and search strategies in Scopus

Domain	Search	Terms
**Intervention (I)**		
**I-1**	#1	TITLE-ABS-KEY(“lifestyle change”)
	#2	TITLE-ABS-KEY(“lifestyle modification”)
	#3	TITLE-ABS-KEY(“lifestyle adaptation”)
	#4	TITLE-ABS-KEY(“lifestyle intervention”)
	#5	TITLE-ABS-KEY (“lifestyle therapy”)
	#6	TITLE-ABS-KEY (“lifestyle treatment”)
	#7	#1 OR #2 OR #3 OR #4 OR #5 OR #6
	#8	TITLE-ABS-KEY (“behavior change”)
	#9	TITLE-ABS-KEY ("behavior modification")
	#10	TITLE-ABS-KEY ("behavior adaptation")
	#11	TITLE-ABS-KEY ("behavior intervention")
	#12	TITLE-ABS-KEY ("behavior therapy")
	#13	TITLE-ABS-KEY ("behavior treatment")
	#14	#8 OR #9 OR #10 OR #11 OR #12 OR #13
	**#15**	**#7 OR #14**
**I-2**	#16	TITLE-ABS-KEY ("non-pharmacological*")
	#17	TITLE-ABS-KEY ("nonpharmacological*")
	**#18**	**#16 OR #17**
**I-3**	#19	TITLE-ABS-KEY ("carbohydrate-restricted diet")
	#20	TITLE-ABS-KEY ("Mediterranean diet")
	#21	TITLE-ABS-KEY ("fat-restricted diet")
	#22	TITLE-ABS-KEY ("reducing diet")
	#23	TITLE-ABS-KEY ("diet therapy")
	#24	#19 OR #20 OR #21 OR #22 OR #23
	#25	TITLE-ABS-KEY ("diet* control")
	#26	TITLE-ABS-KEY ("dietary control")
	#27	#25 OR #26
	#28	TITLE-ABS-KEY ("low glycemic index")
	#29	TITLE-ABS-KEY ("low carbohydrate")

	#30	TITLE-ABS-KEY ("dietary approach to stop hypertension")
	#31	TITLE-ABS-KEY ("healthy eat*")
	#32	TITLE-ABS-KEY ("clean food")
	#33	#28 OR #29 OR #30 OR #31 OR #32
	**#34**	**#24 OR #25 OR #33**
**I-4**	#35	TITLE-ABS-KEY ("physical activity")
	#36	TITLE-ABS-KEY ("exercise")
	#37	TITLE-ABS-KEY ("sport")
	#38	TITLE-ABS-KEY ("workout")
	#39	TITLE-ABS-KEY ("work out")
	#40	#38 OR #39
	#41	TITLE-ABS-KEY ("resistance training")
	#42	TITLE-ABS-KEY ("strength training")
	#43	TITLE-ABS-KEY ("weight training")
	**#44**	**#35 OR #36 OR #37 OR #40 OR #41 OR #42 OR #43**
**I 1-4**	**#45**	**#15 OR #18 OR #34 OR #44**
**Outcome (O)**	#46	TITLE-ABS-KEY ("prevent*")
	#47	TITLE-ABS-KEY ("delay*")
	#48	TITLE-ABS-KEY ("reduc*")
	#49	#46 OR #47OR #48
	#50	TITLE-ABS-KEY ("diabetes mellitus")
	**#51**	**#49 AND #50**
**(I) + (O)**	**#52**	**#45 AND #51**
**Study design**	#53	TITLE-ABS-KEY ("systematic review")
	#54	TITLE-ABS-KEY ("meta analysis")
	**#55**	**#53 OR #54**
**(I) + (O) + Design**	**#56**	**#52AND #55**

Supplementary Table 3. Characteristics of included systematic reviews and meta-analyses

Author, year [Ref]	Population	Intervention	Type of included studies	Age	%Male	Total N	Duration of follow-up (years)	Setting	Country of corresponding author	Funding	COI
Bhupathiraju, 2014 [1]	General population	Low GI and GL	Cohort: 13	NR	0-100	NR	4-14	U.S. :6<br>U.K.: 1<br>European: 4<br>Asian: 2	U.S.	Government	No
Dong, 2011[2]	General population	Low GI or GL	Cohort:13	NR	NR	NR	4-10	U.S. :8<br>U.K.: 1<br>European:1<br>Asian:1<br>Australia, New Zealand: 2	China	Foundation	NR
Greenwood, 2013 [3]	General population	Low GI and GL, or total dietary carbohydrate intake	Cohort: 18	75	0-100	690-91249	4-26	U.S. :9<br>U.K.: 1<br>European: 4<br>Asian: 2<br>Australia, New Zealand :2	U.K.	Government	No
Schwingshackl, 2015[4]	General population	Mediterranean diet	Parallel RCT: 1<br>Cohort: 8	20 - 29 /55-80	0-100	NR	3.2-20	U.S. :3<br>U.K.: 1<br>European: 5	Austria	No	No
Koloverou, 2014[5]	General population, at risk	Mediterranean diet	Parallel RCT: 1<br>Cohort: 9	NR	NR	NR	3.5-14	U.S. :3<br>U.K.: 1<br>European: 6	Greece	No	No
Esposito, 2014[6]	General population	Mediterranean and DASH diet	Cohort: 18	22-44 /27-75	0-100	NR	3.2-23	U.S. :7<br>European: 5<br>Asian: 5	Italy	University	No

Author, year [Ref]	Population	Intervention	Type of included studies	Age	%Male	Total N	Duration of follow-up (years)	Setting	Country of corresponding author	Funding	COI
								Australia, New Zealand :1			
Schwingshackl, 2015[7]	General population	HEI, AHEI, DASH diet	Cohort: 6	30-79	0-100	862-154493	5-24	U.S.:5<br>European: 1	Austria	No	No
Jannasch, 2017[8]	General population	Mediterranean and DASH diet, HEI, AHEI	Parallel RCT: 1<br>Cohort: 47	NR	NR	NR	NR	U.S. :22<br>European: 18<br>Asian: 5<br>Australia, New Zealand :3	Germany	Government	No
Esposito, 2010[9]	General population	Mediterranean, DASH, and AHEI diet	Cohort:10	NR	NR	NR	3.2-23	NR	Italy	NR	No
Maghsoudi, 2016[10]	General population	Healthy diet	Cohort: 10	27-84	0-100	NR	4-23	U.S. :4<br>European :2<br>Asian: 3<br>Australia, New Zealand:1	Iran	No	No
Gilles, 2007[11]	IGT	Diet, PA, Diet+PA	Parallel RCT: 7	25-70	0-57	60-530	NR	US: 1<br>Europe: 1<br>Asia: 4<br>Australia: 1	U.K.	Government	Industry
Stevens, 2015[12]	IFG, IGT	Diet+PA, or pharmacologic interventions,	Parallel RCT: 19	39-65	NR	NR	2.5-9.4	US: 3<br>UK: 3<br>Europe: 5<br>Asia:7	U.K.	Government	Government

Author, year [Ref]	Population	Intervention	Type of included studies	Age	%Male	Total N	Duration of follow-up (years)	Setting	Country of corresponding author	Funding	COI
		or surgical intervention						Australia:1			
Merlotti, 2014[13]	General population	Diet	Parallel RCT: 3	50-72	NR	64 - 18315	1.5-7.7	NR	Italy	NR	No
Carter, 2010[14]	General population	Fruit and vegetables	Cohort: 6	30-74	NR	4304-71346	4.6-23	U.S.: 4<br>European:1<br>Asian :1	U.K.	Government	Government
Cooper, 2012[15]	General population	Fruit and vegetables	Cohort: 7	25-79	NR	NR	4.6-23	U.S.: 4<br>European :2<br>Asian: 1	U.K.	Government	No
Hamer, 2007[16]	General population	Fruit and vegetables, antioxidants (vitamin E, vitamin C, Flavonoids, carotenoids, lycopene)	Cohort:14	18-74	100	895-84360	5-23	U.S.: 10,<br>European: 4	U.K.	Foundation	No
Wang, 2016[17]	Healthy population	Fruit and vegetables	Cohort: 22	25-79	NR	NR	4-24	NR	China	Foundation	No
Li, 2015[18]	General population	Fruit intake	Cohort: 9	40-77	0-100	4304-85104	4.6-25	U.S. :5<br>European :2<br>East Asia:2	China	NR	No
de Munter, 2007[19]	General population	Whole grain	Cohort: 6	21-69 /40-75	NR	NR	6-18	NR	U.S.	Government	No
Ye, 2012[20]	General population	Whole grain	Cohort: 17	NR	NR	NR	4-14	NR	U.S.	Government	No

Author, year [Ref]	Population	Intervention	Type of included studies	Age	%Male	Total N	Duration of follow-up (years)	Setting	Country of corresponding author	Funding	COI
Aune, 2013[21]	General population	Whole grains	Cohort: 16	18-65 /50-79	100	NR	4-23	US:7<br>UK: 1<br>Europe: 5<br>Asia: 2<br>Australia: 1	U.K.	Government	No
Afshin, 2014[22]	General population	Nut or legume	Parallel RCT:1<br>Cohort: 7	NR	0-100	NR	4-23	U.S.: 4<br>European :2<br>Asian: 2	U.S.	Government	No
Tong, 2011[23]	General population	Dairy products	Cohort: 7	39-57	100	NR	5-20	U.S.: 4<br>U.K. :1<br>Asian :2	China	Foundation	No
Aune, 2013b[24]	General population	Dairy products	Cohort:17	20-88	100	640-83779	5-23	U.S. :7<br>European: 6<br>Asian: 2<br>Australia, New Zealand :2	U.K.	Government	No
Chen, 2014[25]	General population	Dairy products	Cohort:14	NR	0-100	NR	5-30	U.S. :6<br>U.K.: 2<br>European: 2<br>Asian: 2<br>Australia, New Zealand :2	U.S.	Government	No
Gijsbers, 2016[26]	Healthy adults	Dairy products	Cohort: 22	36-67	0-100	NR	5-23	U.S. :9<br>U.K.: 3<br>European: 5<br>Asian: 3<br>Australia, New Zealand:2	Netherlands	University	No

Author, year [Ref]	Population	Intervention	Type of included studies	Age	%Male	Total N	Duration of follow-up (years)	Setting	Country of corresponding author	Funding	COI
Muley, 2014[27]	General population	fish, n-3 PUFA	Cohort:16	26-88	0-100	2724-195204	4-23	U.S. :7<br>European: 5<br>Asian: 3<br>Australia, New Zealand :1	India	NR	No
Wu, 2012[28]	General population	fish/seafood, n-3 PUFA	Cohort:16	36-75	100	NR	4-16.7	U.S. :9<br>European: 5<br>Asian :3<br>Australia, New Zealand :1	U.S.	Government, Foundation	Industry
Zhou, 2012[29]	General population	Fish intake, n-3 fatty acid	Cohort:10	26-78	100	2831-116156	4-15	U.S.: 7<br>European:1<br>Asian: 2	China	No	No
Aune, 2009[30]	General population	Meat intake	Cohort: 12	26-46 /55-69	0-100	NR	4-23	U.S. :6<br>U.K.: 1<br>European: 2<br>Asian: 2<br>Australia, New Zealand :1	Norway	Foundation	No
Schwingshackl, 2017b[31]	General population	Olive oil intake	Parallel RCT: 1<br>Cohort: 4	26-45 /55-80	0-100	NR	4.1-22	U.S. :2<br>European: 3	Germany	NR	No
Schwingshackl, 2017[32]	General population	12 food groups	Cohort:88	20-87	0-100	605-401909	NR	NR	Germany	Government	No
Yuan, 2017[33]	General population	Chocolate	Cohort: 5	45-64 /50-79	0-100	NR	4.7-13.3	U.S.: 4<br>Asian: 1	China	NR	No

Author, year [Ref]	Population	Intervention	Type of included studies	Age	%Male	Total N	Duration of follow-up (years)	Setting	Country of corresponding author	Funding	COI
Larsson, 2007[34]	General population	Total, dietary or supplements magnesium	Cohort: 7	NR	100	NR	4-14	U.S.: 6<br>Australia, New Zealand: 1	Sweden	Government	No
Fang, 2016[35]	General population	Dietary and supplement magnesium	Cohort: 25	24.9-61.3	0-100	NR	4-20	U.S. :16<br>European :2<br>Asian: 7	Sweden	University	No
Dong, 2011[36]	General population	Magnesium intake	Cohort: 13	18-30 /55-60	NR	NR	4-20	U.S. :8<br>European: 1<br>Asian: 3<br>Australia, New Zealand :1	China	Foundation	No
Schulze, 2007[37]	General population	Fiber and magnesium intakes	Cohort: 13	26-46 /40-75	0-100	NR	4-18	U.S. 10<br>European :2<br>Australia, New Zealand:1	Germany	Government	No
The InterAct Consortium, 2015[38]	General population	High fiber	Cohort: 19	26 –46 /60 -79	0-100	NR	4-16	U.S. :8<br>U.K.: 1<br>European: 4<br>Asian: 3<br>Australia, New Zealand:3	Netherlands	Government	No
Yao, 2014[39]	General population	Dietary fiber intake	Cohort: 17	26-46 /60-79	0-100	NR	4-24	U.S. :7<br>U.K.: 1<br>European: 6<br>Asian: 1	China	NR	No

Author, year [Ref]	Population	Intervention	Type of included studies	Age	%Male	Total N	Duration of follow-up (years)	Setting	Country of corresponding author	Funding	COI
								Australia, New Zealand:2			
Zheng, 2012[40]	General population	fish, shellfish, n-3PUFA, marine n-3 PUFA, alpha-linolenic acid	Cohort:12	26-79	100	2724-91669	4-18	U.S. :7<br>European: 7<br>Asian: 8<br>Australia, Newzealand:1<br>South America: 1	China	Foundation	No
Xu, 2018[41]	General population	Total flavonoids or flavonoid subclass	Cohort: 8	26-45 /67	NR	NR	4-28	U.S.: 4<br>European: 4	China	NR	No
Hopper, 2011[42]	IFG, IGT	Diet+PA, or pharmacologic interventions	Parallel RCT: 10	45-64	47	37-3304	2.8-6	NR	Australia	Government	No
Selph, 2015[43]	IFG, IGT	Diet+PA, or pharmacologic interventions	Parallel RCT: 9<br>Cluster RCT: 1	45-59	30-72	102-2165	1-6	US: 2<br>UK: 1<br>Europe: 2<br>Asia:5	U.S.	Government	Government
Glechner, 2015[44]	Prediabetes	Diet+PA, or pharmacologic interventions, or surgical intervention	Parallel RCT: 7	44-59	32-72	67-3234	1-6	NR	Austria	Government	Industry
Hemmingsen, 2017[45]	Prediabetes	PA, or Diet+PA	Parallel RCT: 10	45-63	50-100	78-3161	2-6	US: 2<br>Europe: 6<br>Asia: 4	Denmark	No	Industry

Author, year [Ref]	Population	Intervention	Type of included studies	Age	%Male	Total N	Duration of follow-up (years)	Setting	Country of corresponding author	Funding	COI
			Cluster RCT: 2								
Glechner, 2018[46]	Prediabetes	Diet, PA, or Diet+PA	Parallel RCT: 22	44-66	0-96	NR	1-5	NR	Austria	Government	No
Schellenberg, 2013 [47]	High risk (T2DM or MetSyn, PreDM, insulin resistance, syndrome)	Diet+PA plus at least 1 component (smoking cessation, BI)	Parallel RCT: 9	44-85	NR	39-3234	0.5-6	NR	Canada	Government	No
Balk, 2015[48]	PreDM, MetSyn, people at risk with T2DM or CVDs	Diet+PA	Parallel RCT: 3 Cohort: 12	43-65	10 - 86	NR	1-23	NR	U.S.	Government	Government
Merlotti, 2014[49]	Obesity	Diet+PA, or pharmacologic interventions, or surgical intervention	Parallel: 4	51-57	NR	102-1620	0.5-6	NR	Italy	NR	No
Modesti, 2016[50]	Asian population	Diet+PA	Parallel: 8	NR	NR	171-673	1.5-6	Asia: 8	Italy	NR	No

Author, year [Ref]	Population	Intervention	Type of included studies	Age	%Male	Total N	Duration of follow-up (years)	Setting	Country of corresponding author	Funding	COI
Imamura, 2016 [51]	General population	sweetened beverages, artificial sweetened beverages, and fruit juice consumption	Cohort	18–30/ 45–84	NR	NR	3.4-21.1	NR	U.K.	No	No
van Dam, 2005 [52]	General population	Coffee consumption	Cohort	Mean 27/50-74	0 - 100	NR	6-18	NR	U.S.	NR	NR
Raman, 2019 [53]	Hypertension, CVDs	Flavan-3-ols	Cohort: 10	45-69/ 25-75	0 – 100	2915-199980	4 - 18	NR	U.S.	Non-profit organization	No
Uusitupa, 2019 [54]	PreDM and MetSyn	Diet, PA, or Diet+PA	Parallel RCT: 7	NR	NR	102-577	1 – 6	NR	Finland	Foundation	Industry
Yamaoka, 2019 [55]	Overweight, PreDM	Diet, PA, or Diet+PA	Parallel RCT: 19	NR	NR	NR	1-6	NR	Japan	No	No
Li, 2020 [56]	Women with history of GDM	Diet, PA, or Diet+PA	Parallel RCT: 15	NR	NR	NR	NR	NR	China	Government	No

Author, year [Ref]	Population	Intervention	Type of included studies	Age	%Male	Total N	Duration of follow-up (years)	Setting	Country of corresponding author	Funding	COI
Tang, 2020 [57]	General population	Legume and soy intake	Cohort: 15	30-69/ 45-75	0-100	NR	4-18	NR	China	Foundation	No

Abbreviation: Ref, Reference; COI, conflict of interest; CVDs, cardiovascular diseases; DM, diabetes mellitus; GI, glycemic index; GL, glycemic load; LSM, lifestyle modification; MetSyn, Metabolic syndrome; NR, not reported; QoL, Quality of life; PA, Physical activity; PreDM, Pre-diabetes; SSB, sugar sweetened beverages; UC, usual care; GDM: Gestational Diabetes Mellitus.

Supplementary Table 4. Summary results of included systematic reviews and meta-analyses

Author, year [ref]	Comparison	Pooled-RR	95%CI	I^2 (%)	Publication bias	No. of studies	O	E	P-value	ESF
**Dietary factors**										
Dietary intervention: Meta-analysis of RCTs										
Merloti, 2014 [13]	Usual care	0.51	0.39-0.68	29.2	No	3	3	1.58	0.741	No
Stevens, 2015 [12]	Usual care	0.6	0.43-0.82	NR	NR	4	NA	NA	NA	NA
Gilles, 2007 [11]	Usual care	0.67	0.49-0.92	NR	No	3	2	1.4	0.213	No
Yamaoka, 2019 [55]	Usual care	0.71	0.55-0.90	35.7	NR	4	NA	NA	NA	NA
**Dietary pattern**										
Glycemic index										
Bhupathiraju, 2014 [1]	Highest vs lowest	1.12	1.03-1.21	68.5	Yes	13	7	5.73	2.738E-07	**Yes**
Dong, 2011 [2]	Highest vs lowest	1.16	1.06-1.26	50.8	Yes	13	NA	NA	NA	NA
Greenwood 2013 [3]	Per 5 units/D	1.08	1.02-1.15	87	NR	15	NA	NA	NA	NA
**Glycemic Load**										
Bhupathiraju, 2014 [1]	Highest vs lowest	1.12	1.06-1.17	26.4	No	17	NA	NA	NA	NA
Dong, 2011 [2]	Highest vs lowest	1.2	1.11-1.30	34.8	No	12	NA	NA	NA	NA
Greenwood 2013 [3]	Per 20 units/D	1.03	1.00-1.05	54	NR	16	NA	NA	NA	NA
**Mediterranean diet**										
Koloverou, 2014 [5]	Highest vs lowest	0.77	0.66-0.89	NR	NR	10	NA	NA	NA	NA
Esposito, 2014 [6]	Highest vs lowest	0.8	0.68-0.93	NR	NR	6	2	1.64	0.4259	No

Author, year [ref]	Comparison	Pooled-RR	95%CI	I^2 (%)	Publication bias	No. of studies	O	E	P-value	ESF
Schwingshackl, 2015 [4]	Highest vs lowest	0.81	0.73-0.90	55	No	9	6	5.31	0.000005587	**Yes**
Jannasch, 2017 [8]	Highest vs lowest	0.87	0.82-0.93	26	NR	6	NA	NA	NA	NA
DASH diet										
Esposito, 2014 [6]	Highest vs lowest	0.75	0.65-0.83	0	NR	3	NA	NA	NA	NA
Schwingshackl, 2015 [7]	Highest vs lowest	0.79	0.66-0.95	66	NR	4	NA	NA	NA	NA
HEI										
Schwingshackl, 2015 [7]	Highest vs lowest	0.82	0.76-0.88	NR	NR	1	NA	NA	NA	NA
AHEI										
Schwingshackl, 2015 [7]	Highest vs lowest	0.77	0.68-0.86	80	NR	6	4	4.64	0.02	**Yes**
Jannasch, 2017 [8]	Highest vs lowest	0.79	0.70-0.89	88	NR	6	NA	NA	NA	NA
Healthy Diet										
Schwingshackl, 2015 [7]	Highest vs lowest	0.78	0.72-0.8	74	Yes	6	NA	NA	NA	NA
Esposito, 2014 [6]	Highest vs lowest	0.8	0.74-0.86	57	No	18	NA	NA	NA	NA
Jannasch, 2017 [8]	Highest vs lowest	0.84	0.77-0.91	6	NR	7	NA	NA	NA	NA
Maghsoudi, 2016 [10]	Highest vs lowest	0.86	0.82-0.90	2.3	NR	13	6	5.28	4.87E-10	**Yes**
**Food groups**										
Fruit and vegetable (combine)										
Cooper, 2012 [15]	Highest vs lowest	0.93	0.87-1.00	10.1	No	5	0	1.7	0.3696	**Yes**
Wang, 2016 [17]	Highest vs lowest	0.95	0.90-1.02	NR	NR	9	NA	NA	NA	NA

Author, year [ref]	Comparison	Pooled-RR	95%CI	I^2 (%)	Publication bias	No. of studies	O	E	P-value	ESF
Hamer, 2007 [16]	Highest vs lowest	0.96	0.79-1.17	NR	NR	4	1	0.27	0.4521	**Yes**
Carter, 2010 [14]	Highest vs lowest	1	0.92-1.09	0	No	4	NA	NA	NA	NA
Vegetable (total)										
Cooper, 2012 [15]	Highest vs lowest	0.89	0.75-1.03	77.3	No	6	NA	NA	NA	NA
Carter, 2010 [14]	Highest vs lowest	0.91	0.76-1.09	78.1	No	5	NA	NA	NA	NA
Wang, 2016 [17]	Highest vs lowest	0.91	0.82-1.01	57.2	No	NR	NA	NA	NA	NA
Schwingshackl, 2017 [32]	Highest vs lowest	0.95	0.89-1.01	59	No	13	NA	NA	NA	NA
Hamer, 2007 [16]	Highest vs lowest	0.97	0.86-1.09	NR	NR	4	NA	NA	NA	NA
Fruit										
Li, 2015 [18]	Highest vs lowest	0.92	0.86-0.97	37.6	No	9	NA	NA	NA	NA
Schwingshackl, 2017 [32]	Highest vs lowest	0.96	0.93-1.00	29	No	15	NA	NA	NA	NA
Hamer, 2007 [16]	Highest vs lowest	1.01	0.88-1.15	NR	NR	4	NA	NA	NA	NA
Schwingshackl, 2017 [32]	Per 100g/D	0.98	0.97-1.00	21	No	13	NA	NA	NA	NA
Whole grain										
Ye, 2012 [20]	Highest vs lowest	0.74	0.69-0.80	NR	No	6	NA	NA	NA	NA
Aune, 2013 [21]	Highest vs lowest	0.74	0.71-0.78	0	No	9	NA	NA	NA	NA
Schwingshackl, 2017 [32]	Highest vs lowest	0.77	0.71-0.84	86	Yes	13	NA	NA	NA	NA
de Munter, 2007 [19]	Highest vs lowest	0.79	0.72-0.87	68	No	6	NA	NA	NA	NA
Schwingshackl, 2017 [32]	Per 30g/D	0.87	0.82-0.93	91	No	12	NA	NA	NA	NA

Author, year [ref]	Comparison	Pooled-RR	95%CI	I^2 (%)	Publication bias	No. of studies	O	E	P-value	ESF
Refined grain										
Aune, 2013 [21]	Highest vs lowest	0.94	0.82-.109	64	No	6	NA	NA	NA	NA
Schwingshackl, 2017 [32]	Highest vs lowest	1.01	0.92-1.10	54	No	15	NA	NA	NA	NA
Schwingshackl, 2017 [32]	Per 30 g/day	1.01	0.99-1.03	59	No	14	NA	NA	NA	NA
Nuts										
Schwingshackl, 2017 [32]	Highest vs lowest	0.95	0.85-1.05	67	NR	8	NA	NA	NA	NA
Afshin 2014 [22]	Per 4 servings/week	0.87	0.81-0.94	21.8	NR	6	NA	NA	NA	NA
Schwingshackl, 2017 [32]	Per 28 g/day	0.89	0.71-1.12	77	NR	7	NA	NA	NA	NA
Legumes										
Afshin 2014 [22]	Per 4 servings/week	0.78	0.50-1.24	94.6	NR	2	NA	NA	NA	NA
Schwingshackl, 2017 [32]	Per 50 g/day	1.00	0.92-1.09	87	Yes	12	NA	NA	NA	NA
Tang, 2020 [57]	Highest vs lowest	0.95	0.79-1.14	84.8	No	7	NA	NA	NA	NA
Total soy										
Tang, 2020 [57]	Highest vs lowest	0.83	0.68-1.01	90.8	No	9	NA	NA	NA	NA
Soy milk										
Tang, 2020 [57]	Highest vs lowest	0.89	0.71-1.11	91.7	No	5	NA	NA	NA	NA
Tofu										
Tang, 2020 [57]	Highest vs lowest	0.92	0.84-0.99	0	No	4	NA	NA	NA	NA
Soy protein										

Author, year [ref]	Comparison	Pooled-RR	95%CI	I^2 (%)	Publication bias	No. of studies	O	E	P-value	ESF
Tang, 2020 [57]	Highest vs lowest	0.84	0.75-0.95	0	No	3	NA	NA	NA	NA
Soy isoflavones										
Tang, 2020 [57]	Highest vs lowest	0.88	0.81-0.96	37.2	No	6	NA	NA	NA	NA
Dairy products										
High-fat dairy products										
Aune, 2013 [24]	Highest vs lowest	0.96	0.87-1.06	15.8	No	9	NA	NA	NA	NA
Aune, 2013 [24]	Per 200 g/day	0.98	0.94-1.03	7.6	No	9	NA	NA	NA	NA
Gijsbers, 2016 [26]	Per 200 g/day	0.98	0.93-1.04	51.6	NR	13	NA	NA	NA	NA
Low-fat dairy products										
Aune, 2013 [24]	Highest vs lowest	0.83	0.76-0.90	0	No	9	NA	NA	NA	NA
Aune, 2013 [24]	Per 200 g/day	0.91	0.86-0.96	40.2	No	9	NA	NA	NA	NA
Gijsbers, 2016 [26]	Per 200 g/day	0.96	0.92-1.00	68	NR	13	NA	NA	NA	NA
Milk										
Aune, 2013 [24]	Highest vs lowest	0.87	0.70-1.07	70.5	No	7	NA	NA	NA	NA
Aune, 2013 [24] Per 200g/D	Per 200 g/day	0.87	0.72-1.04	93.6	No	7	NA	NA	NA	NA
Cheese										
Aune, 2013 [24]	Highest vs lowest	0.91	0.84-0.98	0	No	8	NA	NA	NA	NA
Aune, 2013 [24]	Per 50 g/day	0.92	0.86-0.99	0	No	8	NA	NA	NA	NA

Author, year [ref]	Comparison	Pooled-RR	95%CI	I^2 (%)	Publication bias	No. of studies	O	E	P-value	ESF
Per 50g/D										
Yogurt										
Aune, 2013 [24]	Highest vs lowest	0.86	0.75-0.98	58.9	No	7	NA	NA	NA	NA
Chen, 2014 [25] Per 1 serving/D	Highest vs lowest	0.82	0.70-0.96	65.3	No	9	NA	NA	NA	NA
Fish										
Schwingshackl, 2017 [32]	Highest vs lowest	1.04	0.95-1.13	76	No	16	NA	NA	NA	NA
Wu, 2012 [28]	Highest vs lowest	1.07	0.94-1.22	77.7	No	13	NA	NA	NA	NA
Zhou, 2012 [29]	Highest vs lowest	1.15	0.98-1.35	79	NR	6	NA	NA	NA	NA
Wu, 2012 [28]	Per 100 g/day	1.12	0.94-1.34	82.9	No	13	NA	NA	NA	NA
Zhou, 2012 [29]	Per 105 g/week	1.04	1.03-1.06	0	NR	3	NA	NA	NA	NA
Schwingshackl, 2017 [32]	Per 100 g/day	1.09	0.93-1.28	84	No	15	NA	NA	NA	NA
Red meat										
Aune, 2009 [30]	Highest vs lowest	1.21	1.07-1.38	58.5	NR	10	NA	NA	NA	NA
Schwingshackl, 2017 [32]	Highest vs lowest	1.21	1.13-1.30	65	No	15	NA	NA	NA	NA
Schwingshackl, 2017 [32]	Per 100 g/day	1.17	1.08-1.26	14	No	14	NA	NA	NA	NA
Processed meat										
Aune, 2009 [30]	Highest vs lowest	1.41	1.25-1.60	53.2	NR	9	NA	NA	NA	NA
Schwingshackl, 2017 [32]	Highest vs lowest	1.27	1.20-1.35	55	Yes	14	NA	NA	NA	NA

Author, year [ref]	Comparison	Pooled-RR	95%CI	I^2 (%)	Publication bias	No. of studies	O	E	P-value	ESF
Schwingshackl, 2017 [32]	Per 50 g/day	1.37	1.22-1.55	88	Yes	14	NA	NA	NA	NA
Eggs										
Schwingshackl, 2017 [32]	Highest vs lowest	1.08	0.95-1.22	69	No	13	NA	NA	NA	NA
Schwingshackl, 2017 [32]	Per 30 g/day	1.08	0.95-1.22	77	No	13	NA	NA	NA	NA
Olive oil										
Schwingshackl, 2017 [31]	Highest vs lowest	0.84	0.77-0.92	22	NR	5	NA	NA	NA	NA
Schwingshackl, 2017 [31]	Per 10 g/day	0.91	0.87-0.95	0	NR	4	NA	NA	NA	NA
Chocolate										
Yuan, 2017 [33]	Highest vs lowest	0.82	0.70-0.96	60	NR	5	NA	NA	NA	NA
Yuan, 2017 [33] Per 2 servings/week	Per 2 servings/week	0.75	0.63-0.89	NR	NR	5	NA	NA	NA	NA
Sugar sweetened beverage										
Schwingshackl, 2017 [32]	Highest vs lowest	1.3	1.20-1.40	34	No	10	NA	NA	NA	NA
Schwingshackl, 2017 [32]	Per 250 ml/day	1.21	1.12-1.31	78	Yes	10	NA	NA	NA	NA
Imamura, 2016 [51]	Per 1 serving/day	1.18	1.09-1.28	89	NR	17	NA	NA	NA	NA
Artificial sugar sweetened beverages										
Imamura, 2016 [51]	Per 1 serving/day	1.25	1.18-1.33	70	NR	10	NA	NA	NA	NA
Coffee consumption										
van Dam, 2005 [52]	Highest vs lowest	0.65	0.54-0.78	NR	No	8	NA	NA	NA	NA

Author, year [ref]	Comparison	Pooled-RR	95%CI	I^2 (%)	Publication bias	No. of studies	O	E	P-value	ESF
**Food Nutrients**										
Total fiber										
Yao, 2014 [39]	Highest vs lowest	0.85	0.77-0.94	53.6	NR	12	NA	NA	NA	NA
The InterAct Consortium, 2015 [38]	Highest vs lowest	0.81	0.73-0.90	61	NR	17	NA	NA	NA	NA
The InterAct Consortium, 2015 [38]	Per 10 g/day	0.91	0.87-0.96	29.4	NR	15	NA	NA	NA	NA
Fruit fiber										
Schulze, 2007 [37]	Highest vs lowest	1.04	0.94-1.15	NR	NR	7	NA	NA	NA	NA
The InterAct Consortium, 2015 [38]	Highest vs lowest	0.96	0.86-1.07	16.9	NR	11	NA	NA	NA	NA
Yao, 2014 [39]	Highest vs lowest	0.95	0.84-1.07	28.5	NR	10	NA	NA	NA	NA
The InterAct Consortium, 2015 [38]	Per 10 g/day	0.95	0.87-1.03	31.1	NR	NR	NA	NA	NA	NA
Vegetable fiber										
Schulze, 2007 [37]	Highest vs lowest	1.04	0.94-1.15	NR	NR	7	NA	NA	NA	NA
The InterAct Consortium, 2015 [38]	Highest vs lowest	0.96	0.86-1.07	48.3	NR	11	NA	NA	NA	NA
Yao, 2014 [39]	Highest vs lowest	0.95	0.84-1.07	59.4	NR	10	NA	NA	NA	NA

Author, year [ref]	Comparison	Pooled-RR	95%CI	I^2 (%)	Publication bias	No. of studies	O	E	P-value	ESF
The InterAct Consortium, 2015 [38]	Per 10 g/day	0.93	0.82-1.05	43.5	NR	NR	NA	NA	NA	NA
Total Magnesium										
Larsson, 2007 [34]	Highest vs lowest	0.83	0.77-0.89	42.5	NR	7	NA	NA	NA	NA
Fang, 2016 [35]	Highest vs lowest	0.83	0.80-0.86	NR	NR	25	NA	NA	NA	NA
Dong, 2011 [36]	Highest vs lowest	0.78	0.73-0.84	42.4	NR	13	NA	NA	NA	NA
Schulze, 2007 [37]	Highest vs lowest	0.77	0.72-0.84	NR	NR	7	NA	NA	NA	NA
Larsson, 2007 [34]	Per 100 mg/day	0.85	0.79-0.92	68.3	No	7	NA	NA	NA	NA
Flavonoids										
Xu, 2018 [41]	Highest vs lowest	0.89	0.82-0.96	63	No	8	NA	NA	NA	NA
Xu, 2018 [41]	Per 300 mg/day	0.95	0.93-0.97	NR	NR	7	NA	NA	NA	NA
Marine N-3 Polyunsaturated Fatty Acids										
Zheng, 2012 [40]	Highest vs lowest	1.07	0.95-1.20	80.8	No	9	NA	NA	NA	NA
Muley, 2014 [27]	Highest vs lowest	0.93	0.75-1.15	70	NR	4	NA	NA	NA	NA
Total Flavan-3-ols										
Raman, 2019 [53]	Highest vs lowest	0.68	0.45-1.02	78.1	NR	2	NA	NA	NA	NA
Monomers										
Raman, 2019 [53]	Highest vs lowest	0.90	0.83 – 0.97	52.8	NR	5	NA	NA	NA	NA
Proanthocyanidin										

Author, year [ref]	Comparison	Pooled-RR	95%CI	I^2 (%)	Publication bias	No. of studies	O	E	P-value	ESF
Raman, 2019 [53]	Highest vs lowest	0.89	0.81 – 0.98	0	NR	3	NA	NA	NA	NA
**Diet + physical activity**										
Gilles, 2007 [11]	Usual care	0.49	0.40 – 0.59	NR	No	7	5	4.43	0.0002	**Yes**
Hopper, 2011 [42]	Usual care	0.52	0.46 - 0.58	NR	NR	4	4	3.91	0.0070	**Yes**
Selph, 2015 [43]	Usual care	0.57	0.43 - 0.70	77	NR	10	7	5.94	0.00002	**Yes**
Stevens, 2015 [12]	Usual care	0.65	0.56 - 0.74	NR	NR	16	9	8.27	8.41E-14	**Yes**
Glechner, 2015 [44]	Usual care	0.63	0.51 - 0.79	0	NR	5	2	2.83	0.0326	**Yes**
Hemmingsen, 2017 [45]	Usual care	0.57	0.50 - 0.64	6	NR	11	4	5.74	0.0003	**Yes**
Glechner, 2018 [46]	Usual care	0.64	0.53 - 0.77	47	NR	11	5	5.12	2.59E-14	**Yes**
Schellenberg, 2013 [47]	Usual care	0.35	0.14 - 0.85	68	NR	4	4	2.5	0.5064	No
Balk, 2015 [48]	Usual care	0.59	0.52 - 0.66	0	NR	15	8	8.93	6.40E-11	**Yes**
Merlotti, 2014 [49]	Usual care	0.44	0.36 - 0.52	1.4	NR	4	3	3.4	0.0419	**Yes**
Modesti, 2016 [50]	Usual care	0.55	0.44 - 0.70	8	NR	8	3	4.7	0.01735	**Yes**
Merlotti, 2014b [13]	Usual care	0.43	0.35 - 0.52	0	NR	12	8	8.06	2.43E-21	**Yes**
Uusitupa, 2019 [54]	Usual care	0.53	0.41-0.67	63.2	NR	7	5	4.78	2.08E-20	**Yes**
Yamaoka, 2019 [55]	Usual care	0.65	0.56-0.75	4.7	NR	19	NA	NA	NA	NA
Li, 2020 [56] (during pregnancy intervention)	Usual care	0.91	0.66-1.25	0	NR	4	NA	NA	NA	NA
Li, 2020 [56] (Postpartum intervention)	Usual care	0.57	0.42-0.78	13.2	NR	10	2	1.91	4.44E-14	**Yes**

Abbreviation: O, observed number of significant; E, expected value of significant finding; CI, confidence interval; ESF, Excess significant finding; NA, Not Applicable; NR, Not reported; RR, risk ratio

Supplementary Table 5. The overlapping among included systematic reviews and meta-analyses

	Number of reviews	Number of included studies	CCA statistic (%)	Degree of overlapping
Dietary pattern	10	73	9.7	Moderate
Food group	20	130	6.2	Slight
Specific nutrients	8	59	11.7	High
Diet + physical activity	12	71	10.6	Moderate
Sugar sweetened beverage	2	18	44.4	Very high

Supplementary Table 6. Detail of intervention in systematic reviews and meta-analyses of RCTs

Author, Year, [Reference]	Diet										Physical activity							Mode of deliver	Setting	BI	Support device	Duration
	A	B	C	D	E	F	G	H	I	Others	J	K	L	M	N	O	Others					
Gilles, 2007 [11]										AHA step II, small portion of meal	√					√	Endurance training, walking	Supervise, Education	Hospital	No	No	NR
Hopper, 2011 [42]									√							√		Supervise, Education	Hospital	No	No	2.8-6 years
Selph, 2015 [43]	√					√		√							√			Supervise, Education	Hospital	Yes	No	1-6 years
Stevens, 2015 [12]									√							√		NR	NR	NR	NR	2.5-9.4 years
Glechner, 2015 [44]									√					√		√		Supervise, Education	Hospital	No	No	1-6 years
Hemmingsen B, 2017 [45]	√			√		√		√			√		√	√	√	√	Endurance training	Supervise, Education	Hospital	Yes	No	2-6 years
Glechner, 2018 [46]									√							√		Supervise, Education	Hospital	Yes	Yes	1-5 years
Schellenberg, 2013 [47]	√		√	√	√	√		√			√		√	√	√	√	Strengthening, Yoga	Supervise, Education	Hospital	Yes	Yes	0.5-6 years
Balk, 2015 [48]	√		√	√		√					√	√	√		√	√		Supervise, Education	Hospital, Web-based	No	No	1-23 years
Merlotti, 2014 [49]									√							√		NR	NR	NR	NR	0.5-16 years
Modesti, 2016 [50]	√		√	√		√		√			√		√			√		Supervise, Education	Hospital	Yes	Yes	1.5-6 years
Merlotti, 2014 [13]	√															√	Nut, olive oil, alcohol reduction	NR	NR	NR	NR	1.5-7.7 years

A = low calories diet; B = very low calories diet; C = low carbohydrate; D = low fat; E = low glycemic index; F = high fiber; G = high protein; H = low sugar; I = just diet control; J = moderately intensity physical activity, K = high intensity physical activity, L = aerobic exercise; M = resistance training; N = leisure activity; O = increased physical activity

BI, Behavioral intervention

Supplementary Table 7. Quality assessment of included studies by AMSTAR 2

Author, year	Domain																Quality
	1	2	3	4	5	6	7	8	9	10	11	12	13	14	15	16	
Gilles, 2007	Yes	No	Yes	Yes	No	Yes	Partial Yes	Partial Yes	Yes	No	Yes	Yes	Yes	Yes	Yes	Yes	low
Hopper, 2011	Yes	No	No	Partial Yes	No	Yes	Partial Yes	Partial Yes	No	No	No	No	No	No	No	Yes	Critically low
Schellenberg, 2013	Yes	Yes	No	Partial Yes	Yes	Yes	Partial Yes	Yes	Yes	No	Yes	No	Yes	Yes	No	Yes	low
Glechner, 2015	Yes	Yes	Yes	Partial Yes	Yes	No	Yes	Partial Yes	Yes	No	Yes	No	No	No	No	Yes	Critically low
Merlotti, 2014	Yes	No	No	Partial Yes	No	No	No	Partial Yes	Yes	No	Yes	No	No	Yes	No	Yes	Critically low
Merlotti, 2014b	Yes	No	No	No	No	No	Yes	No	Yes	No	Yes	No	No	Yes	Yes	Yes	Critically low
Balk, 2015	Yes	No	No	Partial Yes	Yes	Yes	No	Partial Yes	No	No	Yes	No	No	Yes	Yes	Yes	Critically low
Selph, 2015	Yes	Yes	No	No	Yes	Yes	Yes	Yes	Yes	No	Yes	Yes	No	Yes	No	Yes	Critically low
Stevens, 2015	Yes	No	No	Partial Yes	No	No	No	Partial Yes	Partial Yes	No	Yes	No	No	Yes	Yes	Yes	Critically low
Modesti, 2016	Yes	No	No	Partial Yes	No	Yes	No	Yes	No	No	No	No	No	Yes	No	Yes	Critically low
Hemmingsen B, 2017	Yes	No	No	Yes	Yes	Yes	Partial Yes	Yes	Yes	Yes	Yes	No	Yes	Yes	Yes	Yes	low
Pedersen, 2017	Yes	No	No	Partial Yes	Yes	No	Partial Yes	Partial Yes	Partial Yes	No	No	No	No	No	No	Yes	Critically low
Glechner, 2018	Yes	Yes	No	Partial Yes	Yes	Yes	Yes	Partial Yes	Yes	No	Yes	Yes	Yes	Yes	No	Yes	low
Munter, 2007	No	No	No	Partial Yes	No	Yes	No	Partial Yes	No	No	Yes	No	No	Yes	Yes	Yes	Critically low

Author, year	Domain																Quality
	1	2	3	4	5	6	7	8	9	10	11	12	13	14	15	16	
Hamer, 2007	No	No	Yes	Partial Yes	No	Yes	No	No	Yes	No	Yes	No	No	No	Yes	Yes	Critically low
Larsson, 2007	No	No	No	Partial Yes	No	No	No	Partial Yes	No	No	Yes	No	No	No	Yes	Yes	Critically low
Esposito, 2010	No	No	No	Yes	No	Yes	No	No	No	No	Yes	No	No	No	No	No	Critically low
Dong, 2011	No	No	No	No	No	No	No	Partial Yes	No	No	Yes	No	No	No	Yes	Yes	Critically low
Tong, 2011	No	No	No	No	No	No	No	No	No	No	Yes	No	No	Yes	Yes	Yes	Critically low
Cooper, 2012	No	No	No	No	Yes	Yes	No	No	No	No	Yes	No	No	Yes	Yes	Yes	Critically low
Ye, 2012	Yes	No	No	Yes	No	Yes	No	Partial Yes	No	No	Yes	No	No	Yes	Yes	Yes	Critically low
Aune, 2013	No	No	No	No	No	No	No	Partial Yes	No	No	Yes	No	No	Yes	Yes	Yes	Critically low
Greenwood, 2013	No	Yes	No	Yes	No	No	No	No	No	No	Yes	No	No	Yes	Yes	Yes	Critically low
Afshin, 2014	No	No	No	Yes	Yes	Yes	No	Partial Yes	Yes	No	Yes	No	No	Yes	Yes	Yes	Critically low
Bhupathiraju, 2014	Yes	No	No	Yes	No	Yes	No	No	Yes	No	Yes	No	No	Yes	Yes	Yes	Critically low
Chen, 2014	No	Yes	No	Yes	No	No	No	Yes	No	No	No	No	No	Yes	Yes	Yes	Critically low
Esposito, 2014	Yes	Yes	No	Yes	Yes	Yes	No	Yes	Yes	No	Yes	No	No	Yes	Yes	Yes	Critically low
Koloverou, 2014	Yes	No	No	Yes	Yes	No	Yes	Yes	No	No	Yes	No	No	Yes	Yes	Yes	Critically low

Author, year	Domain																Quality
	1	2	3	4	5	6	7	8	9	10	11	12	13	14	15	16	
Yao, 2014	No	No	No	Yes	No	Yes	No	Yes	No	No	Yes	No	No	Yes	Yes	Yes	Critically low
Schwingshackl, 2015	No	No	No	Yes	Yes	Yes	No	Yes	Yes	No	Yes	No	No	Yes	Yes	Yes	Critically low
The InterAct Consortium, 2015	No	No	No	Yes	Yes	Yes	No	Yes	No	No	Yes	No	No	Yes	Yes	Yes	Critically low
Fang, 2016	No	Yes	No	Yes	No	No	No	Yes	Yes	No	Yes	No	No	Yes	Yes	Yes	Critically low
Gijsbers, 2016	No	No	No	Yes	No	No	No	Yes	Yes	No	Yes	No	No	Yes	Yes	Yes	Critically low
Fumiaki Imamura, 2016	Yes	Yes	No	Yes	Yes	Yes	No	Yes	Yes	No	Yes	No	No	Yes	Yes	Yes	Critically low
Maghsoudi, 2016	Yes	No	Yes	Yes	Yes	Yes	No	Yes	Yes	No	Yes	Yes	No	Yes	Yes	Yes	Critically low
Wang, 2016	Yes	No	No	Yes	Yes	Yes	No	Yes	Yes	No	Yes	Yes	Yes	Yes	Yes	Yes	Critically low
Jannasch, 2017	No	No	No	Partial Yes	No	No	No	Yes	Yes	No	Yes	No	No	Yes	Yes	Yes	Critically low
Schwingshackl, 2017b	No	Yes	No	Yes	Yes	No	No	Yes	Yes	No	Yes	No	No	Yes	Yes	Yes	Critically low
Yuan, 2017	No	No	No	Yes	Yes	Yes	No	Yes	Yes	No	Yes	No	No	Yes	Yes	Yes	Critically low
Xu, 2018	No	No	No	Yes	No	Yes	No	Yes	Yes	No	Yes	No	No	Yes	Yes	No	Critically low
van Dam, 2005	No	No	No	No	No	Yes	No	Partial Yes	No	No	Yes	No	No	Yes	Yes	Yes	Critically low
Schulze, 2007	No	No	No	No	No	No	No	Yes	No	No	No	No	No	Yes	No	Yes	Critically low

Author, year	Domain																Quality
	**1**	**2**	**3**	**4**	**5**	**6**	**7**	**8**	**9**	**10**	**11**	**12**	**13**	**14**	**15**	**16**	
Aune, 2009	No	No	No	Yes	No	No	No	Yes	No	No	Yes	No	No	Yes	Yes	Yes	Critically low
Dong, 2011	No	No	No	No	Yes	Yes	No	Yes	No	No	Yes	No	No	Yes	Yes	Yes	Critically low
Zheng, 2012	Yes	Partia l Yes	No	Partial Yes	Yes	No	Partial Yes	Yes	No	No	Yes	No	No	Yes	Yes	Yes	Low
Aune, 2013	Yes	No	No	No	No	No	Yes	Yes	No	No	Yes	No	No	Yes	Yes	Yes	Critically low
Li, 2015	Yes	No	No	Partial Yes	Yes	No	Partial Yes	Yes	Yes	No	Yes	Yes	Yes	Yes	Yes	Yes	Low
Carter, 2010	Yes	No	No	Partial Yes	No	Yes	Partial Yes	Yes	Yes	No	Yes	Yes	Yes	Yes	Yes	Yes	Low
Schwingshackl, 2017	Yes	Yes	No	Partial Yes	No	Yes	Partial Yes	Yes	Yes	No	Yes	Yes	Yes	Yes	Yes	Yes	Moderate
Wu, 2012	Yes	No	No	Partial Yes	Yes	Yes	No	Yes	Yes	No	Yes	No	No	Yes	Yes	Yes	Critically low
Zhou, 2012	No	No	Yes	Yes	Yes	No	No	Yes	Yes	No	Yes	Yes	Yes	Yes	Yes	Yes	Critically low
Muley, 2014	Yes	No	Yes	Yes	Yes	Yes	No	No	Yes	No	No	No	No	Yes	No	Yes	Critically low
Raman, 2019	No	Yes	No	Yes	No	Yes	No	Yes	Yes	No	Yes	No	No	Yes	No	Yes	Critically low
Uusitupa, 2019	Yes	No	No	Yes	No	Yes	No	Yes	Yes	No	Yes	No	No	Yes	Yes	Yes	Critically low
Yamaoka, 2019	Yes	No	No	Partial Yes	Yes	Yes	No	No	Yes	No	Yes	No	Yes	Yes	Yes	Yes	Critically low
Li, 2020	Yes	No	No	Yes	No	No	No	Partial Yes	Yes	No	Yes	Yes	No	No	No	Yes	Critically low

Author, year	Domain																Quality
	1	2	3	4	5	6	7	8	9	10	11	12	13	14	15	16	
Tang, 2020	Yes	Yes	No	Yes	Yes	Yes	No	Yes	Yes	No	Yes	No	No	Yes	Yes	Yes	Critically low

Reference

1. Bhupathiraju SN, Tobias DK, Malik VS, et al. Glycemic index, glycemic load, and risk of type 2 diabetes: results from 3 large US cohorts and an updated meta-analysis. *The American journal of clinical nutrition* 2014;100(1):218-32. doi: 10.3945/ajcn.113.079533 [published Online First: 2014/05/03]
2. Dong JY, Zhang L, Zhang YH, et al. Dietary glycaemic index and glycaemic load in relation to the risk of type 2 diabetes: A meta-analysis of prospective cohort studies. *British Journal of Nutrition* 2011;106(11):1649-54. doi: 10.1017/S000711451100540X
3. Greenwood DC, Threapleton DE, Evans CE, et al. Glycemic index, glycemic load, carbohydrates, and type 2 diabetes: systematic review and dose-response meta-analysis of prospective studies. *Diabetes care* 2013;36(12):4166-71. doi: 10.2337/dc13-0325 [published Online First: 2013/11/23]
4. Schwingshackl L, Missbach B, König J, et al. Adherence to a Mediterranean diet and risk of diabetes: A systematic review and meta-analysis. *Public health nutrition* 2015;18(7):1292-99. doi: 10.1017/S1368980014001542
5. Koloverou E, Esposito K, Giugliano D, et al. The effect of Mediterranean diet on the development of type 2 diabetes mellitus: a meta-analysis of 10 prospective studies and 136,846 participants. *Metabolism: clinical and experimental* 2014;63(7):903-11. doi: 10.1016/j.metabol.2014.04.010 [published Online First: 2014/06/17]
6. Esposito K, Chiodini P, Maiorino MI, et al. Which diet for prevention of type 2 diabetes? A meta-analysis of prospective studies. *Endocrine* 2014;47(1):107-16. doi: 10.1007/s12020-014-0264-4 [published Online First: 2014/04/20]
7. Schwingshackl L, Hoffmann G. Diet quality as assessed by the Healthy Eating Index, the Alternate Healthy Eating Index, the Dietary Approaches to Stop Hypertension score, and health outcomes: a systematic review and meta-analysis of cohort studies. *J Acad Nutr Diet* 2015;115(5):780-800.e5. doi: 10.1016/j.jand.2014.12.009
8. Jannasch F, Kröger J, Schulze MB. Dietary patterns and Type 2 diabetes: A systematic literature review and meta- analysis of prospective studies. *Journal of Nutrition* 2017;147(6):1174-82. doi: 10.3945/jn.116.242552
9. Esposito K, Kastorini CM, Panagiotakos DB, et al. Prevention of type 2 diabetes by dietary patterns: a systematic review of prospective studies and meta-analysis. *Metabolic syndrome and related disorders* 2010;8(6):471-6. doi: 10.1089/met.2010.0009 [published Online First: 2010/10/21]
10. Maghsoudi Z, Ghiasvand R, Salehi-Abargouei A. Empirically derived dietary patterns and incident type 2 diabetes mellitus: a systematic review and meta-analysis on prospective observational studies. *Public health nutrition* 2016;19(2):230-41. doi: 10.1017/s1368980015001251 [published Online First: 2015/04/29]

11. Gillies CL, Abrams KR, Lambert PC, et al. Pharmacological and lifestyle interventions to prevent or delay type 2 diabetes in people with impaired glucose tolerance: systematic review and meta-analysis. *BMJ (Clinical research ed)* 2007;334(7588):299. doi: 10.1136/bmj.39063.689375.55 [published Online First: 2007/01/24]
12. Stevens JW, Khunti K, Harvey R, et al. Preventing the progression to type 2 diabetes mellitus in adults at high risk: a systematic review and network meta-analysis of lifestyle, pharmacological and surgical interventions. *Diabetes research and clinical practice* 2015;107(3):320-31. doi: 10.1016/j.diabres.2015.01.027 [published Online First: 2015/02/02]
13. Merlotti C, Morabito A, Pontiroli AE. Prevention of type 2 diabetes; a systematic review and meta-analysis of different intervention strategies. *Diabetes, obesity & metabolism* 2014;16(8):719-27. doi: 10.1111/dom.12270 [published Online First: 2014/01/31]
14. Carter P, Gray LJ, Troughton J, et al. Fruit and vegetable intake and incidence of type 2 diabetes mellitus: systematic review and meta-analysis. *BMJ (Clinical research ed)* 2010;341:c4229. doi: 10.1136/bmj.c4229 [published Online First: 2010/08/21]
15. Cooper AJ, Forouhi NG, Ye Z, et al. Fruit and vegetable intake and type 2 diabetes: EPIC-InterAct prospective study and meta-analysis. *European journal of clinical nutrition* 2012;66(10):1082-92. doi: 10.1038/ejcn.2012.85 [published Online First: 2012/08/03]
16. Hamer M, Chida Y. Intake of fruit, vegetables, and antioxidants and risk of type 2 diabetes: systematic review and meta-analysis. *Journal of hypertension* 2007;25(12):2361-9. doi: 10.1097/HJH.0b013e3282efc214 [published Online First: 2007/11/07]
17. Wang PY, Fang JC, Gao ZH, et al. Higher intake of fruits, vegetables or their fiber reduces the risk of type 2 diabetes: A meta-analysis. *Journal of diabetes investigation* 2016;7(1):56-69. doi: 10.1111/jdi.12376
18. Li S, Miao S, Huang Y, et al. Fruit intake decreases risk of incident type 2 diabetes: an updated meta-analysis. *Endocrine* 2015;48(2):454-60. doi: 10.1007/s12020-014-0351-6 [published Online First: 2014/07/31]
19. de Munter JS, Hu FB, Spiegelman D, et al. Whole grain, bran, and germ intake and risk of type 2 diabetes: a prospective cohort study and systematic review. *PLoS medicine* 2007;4(8):e261. doi: 10.1371/journal.pmed.0040261 [published Online First: 2007/09/01]
20. Ye EQ, Chacko SA, Chou EL, et al. Greater whole-grain intake is associated with lower risk of type 2 diabetes, cardiovascular disease, and weight gain. *The Journal of nutrition* 2012;142(7):1304-13. doi: 10.3945/jn.111.155325 [published Online First: 2012/06/01]
21. Aune D, Norat T, Romundstad P, et al. Whole grain and refined grain consumption and the risk of type 2 diabetes: a systematic review and dose-response meta-analysis of cohort studies. *European journal of epidemiology*

2013;28(11):845-58. doi: 10.1007/s10654-013-9852-5 [published Online First: 2013/10/26]

22. Afshin A, Micha R, Khatibzadeh S, et al. Consumption of nuts and legumes and risk of incident ischemic heart disease, stroke, and diabetes: a systematic review and meta-analysis. *The American journal of clinical nutrition* 2014;100(1):278-88. doi: 10.3945/ajcn.113.076901 [published Online First: 2014/06/06]
23. Tong X, Dong JY, Wu ZW, et al. Dairy consumption and risk of type 2 diabetes mellitus: a meta-analysis of cohort studies. *European journal of clinical nutrition* 2011;65(9):1027-31. doi: 10.1038/ejcn.2011.62 [published Online First: 2011/05/12]
24. Aune D, Norat T, Romundstad P, et al. Dairy products and the risk of type 2 diabetes: a systematic review and dose-response meta-analysis of cohort studies. *The American journal of clinical nutrition* 2013;98(4):1066-83. doi: 10.3945/ajcn.113.059030 [published Online First: 2013/08/16]
25. Chen M, Sun Q, Giovannucci E, et al. Dairy consumption and risk of type 2 diabetes: 3 cohorts of US adults and an updated meta-analysis. *BMC medicine* 2014;12:215. doi: 10.1186/s12916-014-0215-1 [published Online First: 2014/11/26]
26. Gijsbers L, Ding EL, Malik VS, et al. Consumption of dairy foods and diabetes incidence: a dose-response meta-analysis of observational studies. *The American journal of clinical nutrition* 2016;103(4):1111-24. doi: 10.3945/ajcn.115.123216 [published Online First: 2016/02/26]
27. Muley A, Muley P, Shah M. ALA, fatty fish or marine n-3 fatty acids for preventing DM?: a systematic review and meta-analysis. *Current diabetes reviews* 2014;10(3):158-65. [published Online First: 2014/05/16]
28. Wu JH, Micha R, Imamura F, et al. Omega-3 fatty acids and incident type 2 diabetes: a systematic review and meta-analysis. *The British journal of nutrition* 2012;107 Suppl 2:S214-27. doi: 10.1017/s0007114512001602 [published Online First: 2012/05/25]
29. Zhou Y, Tian C, Jia C. Association of fish and n-3 fatty acid intake with the risk of type 2 diabetes: a meta-analysis of prospective studies. *The British journal of nutrition* 2012;108(3):408-17. doi: 10.1017/s0007114512002036 [published Online First: 2012/08/04]
30. Aune D, Ursin G, Veierod MB. Meat consumption and the risk of type 2 diabetes: a systematic review and meta-analysis of cohort studies. *Diabetologia* 2009;52(11):2277-87. doi: 10.1007/s00125-009-1481-x [published Online First: 2009/08/08]
31. Schwingshackl L, Lampousi AM, Portillo MP, et al. Olive oil in the prevention and management of type 2 diabetes mellitus: a systematic review and meta-analysis of cohort studies and intervention trials. *Nutrition & diabetes* 2017;7(4):e262. doi: 10.1038/nutd.2017.12 [published Online First: 2017/04/11]

32. Schwingshackl L, Hoffmann G, Lampousi AM, et al. Food groups and risk of type 2 diabetes mellitus: a systematic review and meta-analysis of prospective studies. *European journal of epidemiology* 2017;32(5):363-75. doi: 10.1007/s10654-017-0246-y [published Online First: 2017/04/12]
33. Yuan S, Li X, Jin Y, et al. Chocolate consumption and risk of coronary heart disease, stroke, and diabetes: A meta-analysis of prospective studies. *Nutrients* 2017;9(7) doi: 10.3390/nu9070688
34. Larsson SC, Wolk A. Magnesium intake and risk of type 2 diabetes: A meta-analysis. *Journal of internal medicine* 2007;262(2):208-14. doi: 10.1111/j.1365-2796.2007.01840.x
35. Fang X, Han H, Li M, et al. Dose-Response Relationship between Dietary Magnesium Intake and Risk of Type 2 Diabetes Mellitus: A Systematic Review and Meta-Regression Analysis of Prospective Cohort Studies. *Nutrients* 2016;8(11) doi: 10.3390/nu8110739 [published Online First: 2016/11/22]
36. Dong JY, Xun P, He K, et al. Magnesium intake and risk of type 2 diabetes: meta-analysis of prospective cohort studies. *Diabetes care* 2011;34(9):2116-22. doi: 10.2337/dc11-0518 [published Online First: 2011/08/27]
37. Schulze MB, Schulz M, Heidemann C, et al. Fiber and magnesium intake and incidence of type 2 diabetes: a prospective study and meta-analysis. *Archives of internal medicine* 2007;167(9):956-65. doi: 10.1001/archinte.167.9.956 [published Online First: 2007/05/16]
38. The InterAct Consortium. Dietary fibre and incidence of type 2 diabetes in eight European countries: the EPIC-InterAct Study and a meta-analysis of prospective studies. *Diabetologia* 2015;58(7):1394-408. doi: 10.1007/s00125-015-3585-9 [published Online First: 2015/05/30]
39. Yao B, Fang H, Xu W, et al. Dietary fiber intake and risk of type 2 diabetes: a dose-response analysis of prospective studies. *European journal of epidemiology* 2014;29(2):79-88. doi: 10.1007/s10654-013-9876-x [published Online First: 2014/01/07]
40. Zheng JS, Huang T, Yang J, et al. Marine N-3 polyunsaturated fatty acids are inversely associated with risk of type 2 diabetes in Asians: a systematic review and meta-analysis. *PloS one* 2012;7(9):e44525. doi: 10.1371/journal.pone.0044525 [published Online First: 2012/09/18]
41. Xu H, Luo J, Huang J, et al. Flavonoids intake and risk of type 2 diabetes mellitus: A meta-analysis of prospective cohort studies. *Medicine* 2018;97(19):e0686. doi: 10.1097/md.0000000000010686 [published Online First: 2018/05/10]
42. Hopper I, Billah B, Skiba M, et al. Prevention of diabetes and reduction in major cardiovascular events in studies of subjects with prediabetes: meta-analysis of randomised controlled clinical trials. *Eur J Cardiovasc Prev Rehabil* 2011;18(6):813-23. doi: 10.1177/1741826711421687

43. Selph S, Dana T, Blazina I, et al. Screening for type 2 diabetes mellitus: a systematic review for the U.S. Preventive Services Task Force. *Annals of internal medicine* 2015;162(11):765-76. doi: 10.7326/m14-2221 [published Online First: 2015/04/14]
44. Glechner A, Harreiter J, Gartlehner G, et al. Sex-specific differences in diabetes prevention: a systematic review and meta-analysis. *Diabetologia* 2014;58(2):242-54. doi: 10.1007/s00125-014-3439-x
45. Hemmingsen B, Gimenez-Perez G, Mauricio D, et al. Diet, physical activity or both for prevention or delay of type 2 diabetes mellitus and its associated complications in people at increased risk of developing type 2 diabetes mellitus. *Cochrane Database of Systematic Reviews* 2017;2017(12) doi: 10.1002/14651858.CD003054.pub4
46. Glechner A, Keuchel L, Affengruber L, et al. Effects of lifestyle changes on adults with prediabetes: A systematic review and meta-analysis. *Primary care diabetes* 2018 doi: https://doi.org/10.1016/j.pcd.2018.07.003
47. Schellenberg ES, Dryden DM, Vandermeer B, et al. Lifestyle interventions for patients with and at risk for type 2 diabetes: a systematic review and meta-analysis. *Ann Intern Med* 2013;159(8):543-51. doi: 10.7326/0003-4819-159-8-201310150-00007
48. Balk EM, Earley A, Raman G, et al. Combined Diet and Physical Activity Promotion Programs to Prevent Type 2 Diabetes Among Persons at Increased Risk: A Systematic Review for the Community Preventive Services Task Force. *Annals of internal medicine* 2015;163(6):437-51. doi: 10.7326/m15-0452 [published Online First: 2015/07/15]
49. Merlotti C, Morabito A, Ceriani V, et al. Prevention of type 2 diabetes in obese at-risk subjects: a systematic review and meta-analysis. *Acta diabetologica* 2014;51(5):853-63. doi: 10.1007/s00592-014-0624-9
50. Modesti PA, Galanti G, Cala' P, et al. Lifestyle interventions in preventing new type 2 diabetes in Asian populations. *Internal and emergency medicine* 2016;11(3):375-84. doi: 10.1007/s11739-015-1325-2
51. Imamura F, O'Connor L, Ye Z, et al. Consumption of sugar sweetened beverages, artificially sweetened beverages, and fruit juice and incidence of type 2 diabetes: systematic review, meta-analysis, and estimation of population attributable fraction. *British journal of sports medicine* 2016;50(8):496-504. doi: 10.1136/bjsports-2016-h3576rep [published Online First: 2016/04/06]
52. van Dam RM, Hu FB. Coffee consumption and risk of type 2 diabetes: a systematic review. *Jama* 2005;294(1):97-104. doi: 10.1001/jama.294.1.97 [published Online First: 2005/07/07]
53. Raman G, Avendano EE, Chen S, et al. Dietary intakes of flavan-3-ols and cardiometabolic health: systematic review and meta-analysis of randomized trials and prospective cohort studies. *Am J Clin Nutr* 2019;110(5):1067-78. doi: 10.1093/ajcn/nqz178 [published Online First: 2019/09/11]

54. Uusitupa M, Khan TA, Viguiliouk E, et al. Prevention of Type 2 Diabetes by Lifestyle Changes: A Systematic Review and Meta-Analysis. *Nutrients* 2019;11(11) doi: 10.3390/nu11112611 [published Online First: 2019/11/07]
55. Yamaoka K, Nemoto A, Tango T. Comparison of the Effectiveness of Lifestyle Modification with Other Treatments on the Incidence of Type 2 Diabetes in People at High Risk: A Network Meta-Analysis. *Nutrients* 2019;11(6) doi: 10.3390/nu11061373 [published Online First: 2019/06/30]
56. Li N, Yang Y, Cui D, et al. Effects of lifestyle intervention on long-term risk of diabetes in women with prior gestational diabetes: A systematic review and meta-analysis of randomized controlled trials. *Obes Rev* 2020 doi: 10.1111/obr.13122
57. Tang J, Wan Y, Zhao M, et al. Legume and soy intake and risk of type 2 diabetes: a systematic review and meta-analysis of prospective cohort studies. *Am J Clin Nutr* 2020;111(3):677-88. doi: 10.1093/ajcn/nqz338 [published Online First: 2020/01/10]
